# Evaluating outcomes of renal angioembolization: A retrospective study

**DOI:** 10.6026/973206300214869

**Published:** 2025-12-15

**Authors:** Rana Pratap Singh, Prashant Kumar, Prabin Kumar Shrivastava

**Affiliations:** 1 Department of Urology, Rajendra Institute of Medical Sciences (RIMS), Ranchi, Jharkhand, India; 2 Department of Cardiology, Rajendra Institute of Medical Sciences (RIMS), Ranchi, Jharkhand, India

**Keywords:** Renal Angioembolization (RAE), renal trauma, iatrogenic injury, interventional cardiology and urology, minimally invasive surgery, angiomyolipoma, renal artery embolization

## Abstract

Acute renal hemorrhage requires rapid, nephron-sparing management and renal angioembolization (RAE) has emerged as a minimally invasive alternative to surgery, though outcome data from varied clinical settings remain limited. Therefore, it is of interest to evaluate 79 patients who underwent RAE between January 2018 and January 2023, assessing technical and clinical success, complications and renal function changes. High-grade renal trauma (45.6%) and iatrogenic injury (29.1%) were the leading indications. RAE achieved a technical success rate of 98.7% and clinical success in 93.7% of patients, with post-embolization syndrome being the most common complication (26.6%) and major complications rare (1.3%). Thus, we show that RAE is a highly effective, safe and nephron-sparing first-line treatment for acute renal hemorrhage.

## Background:

The therapeutic landscape for acute renal hemorrhage has been revolutionized by the progression of endovascular techniques. Conditions that historically mandated emergency laparotomy and nephrectomy, such as high-grade renal trauma, ruptured renal tumors, or vascular complications from percutaneous procedures, are now increasingly managed with minimally invasive approaches [1]. Open surgery, while effective for hemostasis, is associated with substantial morbidity, including the definitive loss of renal parenchyma, prolonged hospitalization and significant postoperative pain [2]. Renal angioembolization (RAE) emerged in the 1970s, initially as a palliative measure for advanced renal malignancies, but has since evolved into a primary therapeutic modality for a wide spectrum of emergent conditions [3]. This procedure involves precise, catheter-directed occlusion of bleeding renal arteries, offering the distinct advantages of preserving healthy renal tissue, reducing procedural morbidity and facilitating quicker patient recovery [4, 5]. The kidney is the third most frequently injured solid organ in abdominal trauma, making trauma a leading indication for RAE [6]. The adoption of non-operative management (NOM) for even high-grade (American Association for the Surgery of Trauma [AAST] grades IV-V) renal injuries has been largely facilitated by the availability of RAE, which serves as a crucial adjunct for controlling active bleeding in hemodynamically unstable patients, with reported success rates often exceeding 90% [7, 8]. Concurrently, the proliferation of urological procedures like percutaneous nephrolithotomy (PCNL) and renal biopsy has led to an increased incidence of iatrogenic vascular injuries, such as pseudoaneurysms and arteriovenous fistulas, which are elegantly and effectively managed with superselective embolization [9]. Despite the established role of RAE, ongoing analysis of clinical outcomes from high-volume tertiary centers is vital. Such studies provide real-world evidence of efficacy, document complication rates in complex patient populations and help refine patient selection and procedural techniques [10]. While the procedure's efficacy is well-supported by international literature, there remains a need for contemporary data from institutions in diverse geographical and healthcare settings. Therefore, it is of interest to evaluate the procedure's technical and clinical success rates across various indications and to systematically characterize the spectrum and frequency of associated complications.

## Materials and Methods:

## Study design and setting:

This single-center, retrospective cohort study was performed at the Department of Cardiology and Urology at Rajendra Institute of Medical Sciences (RIMS), Ranchi, Jharkhand, a major tertiary referral hospital in India. The study was granted ethical clearance by the Institutional Ethics Committee (IEC Reg. No - ECR/769/INST/JH/2015/RR-21). The need for individual informed consent was waived due to the study's retrospective nature.

## Study population:

We reviewed the records of all consecutive patients aged 16 and older who underwent RAE for acute renal hemorrhage between January 1, 2023 and January 31, 2025. Patients were identified using procedural codes from the hospital's electronic health records (EHR) and the cardiology and urology department's Picture Archiving and Communication System (PACS).

## Inclusion criteria:

[1] Age ≥ 16 years.

[2] Procedure performed for acute renal hemorrhage resulting from trauma, iatrogenic injury, ruptured renal neoplasms, or vascular malformations.

[3] Complete medical and radiological records available for review.

## Exclusion criteria:

[1] Age < 16 years.

[2] RAE performed for non-emergent indications (e.g., elective preoperative tumor devascularization).

[3] Incomplete or missing clinical or procedural data.

[4] Patients with pre-existing end-stage renal disease on dialysis.

An initial search identified 92 patient records. After review, 13 patients were excluded (7 for incomplete data, 4 for elective indications and 2 under the age of 16), yielding a final study cohort of 79 patients.

## Procedural technique:

All RAE procedures were conducted by one of three board-certified interventional cardiologists. The standard technique involved obtaining arterial access via the common femoral artery using the Seldinger method under local anesthesia. A 5-French vascular sheath was introduced. Following an initial abdominal aortogram, a 5-French selective catheter was used to engage the ostium of the affected renal artery. Digital subtraction angiography (DSA) was then performed to identify the source of bleeding, typically visualized as active contrast extravasation, a pseudoaneurysm, or an arteriovenous fistula. A microcatheter (2.7-French or smaller) was advanced coaxially to achieve a superselective position in the target sub-segmental artery. The choice of embolic agent was determined by the interventional radiologist based on the angiographic findings and vessel anatomy. Materials used included absorbable gelatin sponge (Gelfoam), metallic microcoils, polyvinyl alcohol (PVA) particles and N-butyl cyanoacrylate (NBCA) glue. The procedural endpoint was defined as the complete angiographic cessation of hemorrhage with maximal preservation of blood flow to the non-affected renal parenchyma.

## Data collection and outcome variables:

A standardized data collection form in Microsoft Excel was used to extract the following information from the EHR and PACS:

[1] Demographics and clinical data: Age, sex, indication for RAE, pre-procedural laboratory values (hemoglobin, serum creatinine).

[2] Procedural data: Angiographic findings, embolic agent(s) used, procedure duration.

[3] Outcome measures:

1) Technical success: Successful catheterization and deployment of embolic agent at the target site, resulting in complete angiographic cessation of hemorrhage.

2) Clinical success: Hemodynamic stabilization and resolution of gross hematuria, with no need for further blood transfusions, repeat embolization, or surgical intervention for bleeding within 30 days.

3) Complications: Any adverse event occurring within 30 days post-procedure, categorized as minor (e.g., post-embolization syndrome, minor access-site hematoma) or major (e.g., non-target embolization, renal abscess, access-site pseudoaneurysm requiring intervention, significant renal function impairment). A significant impairment in renal function was defined as a >50% increase in serum creatinine from baseline.

## Statistical analysis:

Data analysis was performed using SPSS (Version 25.0, IBM Corp). Continuous variables are reported as mean ± standard deviation (SD), while categorical data are presented as frequencies and percentages (%). Pre- and post-procedural serum creatinine values were compared using a paired t-test, with a p-value < 0.05 considered statistically significant.

## Results:

The study included 79 patients with a mean age of 48 ± 12 years (range: 17-78 years). There was a male predominance, with 50 males (63.3%) and 29 females (36.7%). Renal trauma was the most frequent indication for RAE, accounting for 36 cases (45.6%). Iatrogenic injury was the second most common cause (n=23, 29.1%). Other significant indications included ruptured angiomyolipoma (n=14, 17.7%) and bleeding from renal cell carcinoma (n=5, 6.3%). Baseline patient characteristics are summarized in Table 1 (see PDF). The most common angiographic finding indicating the source of hemorrhage was active contrast extravasation, seen in 39 patients (49.4%). A pseudoaneurysm was identified in 28 patients (35.4%) and an arteriovenous fistula was present in 9 patients (11.4%). Metallic microcoils were the most frequently utilized embolic agent, employed in 42 procedures (53.2%). Gelfoam slurry was used in 26 cases (32.9%). Procedural details are outlined in Table 2 (see PDF). Technical success was achieved in 78 of 79 procedures (98.7%). One procedure was deemed a technical failure due to the inability to selectively catheterize a distal, tortuous bleeding vessel. Overall clinical success was achieved in 74 patients (93.7%). Five patients (6.3%) experienced clinical failure. Among these, three had recurrent bleeding that was successfully managed with a repeat RAE procedure; one patient required an emergency nephrectomy for uncontrolled hemorrhage and one patient died from multi-organ failure attributed to the severity of the initial hemorrhagic shock. Complications are detailed in Table 3 (see PDF). The most common adverse event was post-embolization syndrome (PES), which occurred in 21 patients (26.6%) and was successfully managed with conservative measures. Major complications were rare. One patient (1.3%) developed a significant rise in serum creatinine and another (1.3%) had a procedure-related arterial dissection that did not require intervention. No instances of renal abscess or major access-site complications were recorded.

## Discussion:

This retrospective analysis of 79 cases of renal angioembolization reinforces its status as a cornerstone in the management of acute renal hemorrhage. Our study found a high clinical success rate of 93.7%, a figure that aligns closely with outcomes reported in major international series, where success rates typically range between 85% and 98% [11, 12]. This result highlights the procedure's reliability in achieving durable hemostasis and preventing the need for more invasive surgical interventions. The indications for RAE in our cohort mirror the case mix expected at a tertiary trauma and referral center. The predominance of renal trauma is consistent with the global shift towards NOM for high-grade injuries, where RAE is an indispensable tool for managing active bleeding [7, 13]. Our findings also reflect the growing burden of iatrogenic injuries, a direct consequence of the increasing number of percutaneous renal procedures performed worldwide. In this context, RAE provides a targeted, highly effective solution with minimal morbidity [9, 14]. Our technical success rate of 98.7% is a testament to modern interventional cardiology and urology capabilities, including advanced imaging guidance, sophisticated microcatheter systems and a versatile array of embolic agents. This technical precision allows for the superselective targeting of bleeding vessels, which is paramount to the procedure's primary goal: stopping hemorrhage while preserving as much healthy renal parenchyma as possible [15]. The safety profile of RAE in our study was excellent. The most frequent adverse event was Post-Embolization Syndrome (PES), occurring in 26.6% of patients. This incidence is consistent with published rates and is recognized as an expected inflammatory response to ischemic tissue, not a true complication [16]. Importantly, major complications were exceedingly rare. The preservation of renal function is a key concern and our data are reassuring, with only one patient (1.3%) experiencing a significant post-procedural rise in creatinine. This low rate demonstrates that with meticulous, superselective technique, RAE has a minimal impact on overall renal function, a finding supported by other large series [17]. The strengths of our study include its detailed analysis of a heterogenous patient population treated with a standardized approach at a single institution. This provides valuable real-world data from a specific healthcare environment. However, the study's limitations must be acknowledged. First, its retrospective design introduces potential for information bias. Second, the modest sample size of 79 patients may limit the statistical power to detect associations or predictors of failure and restricts the generalizability of our findings. A larger cohort would be needed to draw more definitive conclusions about rare complications. Finally, our follow-up was primarily focused on the short-term (30-day) period, which precludes an assessment of long-term outcomes like the incidence of post-embolization hypertension or chronic kidney disease.

## Conclusion:

Renal angioembolization consistently proves to be a safe and highly effective option for managing acute renal hemorrhage while preserving renal function. Its excellent clinical success rate and low incidence of major complications support its use as a first-line, minimally invasive therapy. Broader multi-center research is needed to strengthen long-term evidence and refine patient selection.
